# Ambulatory antibiotic prescribing for children in a practice research network

**DOI:** 10.1017/ash.2023.272

**Published:** 2023-09-29

**Authors:** Lauren Mitchell, Matthew Kronman, Allison Cole, Nicole Poole

## Abstract

**Background:** Most antibiotic use occurs in ambulatory settings. Antibiotic prescribing for children living in the United States in medically underserved areas or in populations is not well understood.

**Objective:** To characterize antibiotic prescribing for children in a practice-based research network (PBRN).

**Design and Methods:** In this retrospective cohort study, we characterized oral antibiotic prescribing in a large PBRN. Patients aged 0–17 years with at least 1 in-person visit between January 1, 2014, and December 31, 2018, at 1 of 25 primary-care clinics located within the WWAMI (Washington, Wyoming, Alaska, Montana, and Idaho) region of the Practice and Research Network (WPRN) were included. Data were extracted from DataQUEST, a centralized data repository from included primary-care clinics. Encounters for wellness visits or those lacking a diagnosis code and patients with complex chronic conditions were excluded. Diagnoses were categorized using *International Classification of Disease, Ninth Revision* (ICD-9) and ICD-10 codes. Oral antibiotics prescribed within 3 days of an encounter were associated with that encounter. Demographic data included age, sex, race, and ethnicity. Antibiotic appropriateness was determined using a previously published 3-tiered classification system using diagnosis codes as always, sometimes, or never appropriate. Patient-level data (ZIP codes) were used to designate medically underserved areas (MUAs) and medically underserved populations (MUPs). Antibiotic prescribing was then analyzed within these groups. **Results:** In total, 37,314 patients across 206,845 encounters were included, of which 34,601 encounters (17%) resulted in antibiotic prescription (Table 1). Of those, appropriateness data were available for 34,286 (99%). Of the antibiotics prescribed, 14% were always appropriate, 57% were sometimes appropriate, and 27% were never appropriate (1% missing). In total, 64% and 35% of encounters occurred with patients from an MUA and MUP, respectively. **Conclusions:** Targets to improve oral antibiotic prescribing for children in a large PBRN include antibiotic prescribing for diagnoses that never require an antibiotic. Larger comparative studies may focus on the role (if any) that MUA/MUP has on antibiotic prescribing.

**Figure:**
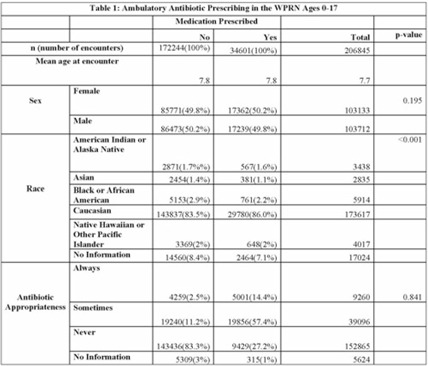

**Disclosures:** None

